# Identifying Gaps in Asthma Education, Health Promotion, and Social Support for Mi’kmaq Families in Unama’ki (Cape Breton), Nova Scotia, Canada

**DOI:** 10.5888/pcd9.120039

**Published:** 2012-08-16

**Authors:** Rob Watson, Heather Castleden, Tui’kn Partnership, Jeffrey Masuda, Malcolm King, Miriam Stewart

**Affiliations:** Author Affiliations: Rob Watson, Dalhousie University, Halifax, Nova Scotia, Canada; Tui’kn Partnership (comprising health directors from the 5 Mi’kmaq communities in Unama’ki, [Cape Breton], Nova Scotia, Canada: Elaine Allison, Darlene Anganis, Jennifer MacDonald, Sharon Rudderham, and Laurie Touesnard); Jeffrey Masuda, University of Manitoba, Winnipeg, Manitoba; Malcolm King, Miriam Stewart, University of Alberta, Edmonton, Canada.

## Abstract

**Introduction:**

Asthma is the most common chronic condition affecting Aboriginal youth aged 8 to 12 years in Canada. Research investigating psychosocial challenges associated with asthma is limited. This study examines support resources, support-seeking strategies, support and education needs, and intervention preferences of Aboriginal youth with asthma and their caregivers in an effort to encourage community-wide, health-promoting behaviors.

**Methods:**

We employed a community-based participatory research design to conduct interviews with 21 youths aged 8 to 12 years and 17 caregivers from 5 Mi’kmaq communities in Unama’ki (Cape Breton) Nova Scotia, Canada. After conducting interviews that explored existing and desired social, educational, and health support in participating communities, we held a 2-day asthma camp to engage participants in asthma education, social support networking, and cultural activities. At the camp, we collected data through participant observation, sharing circles, focus groups, and youth drawings of their experiences living with asthma.

**Results:**

Our study yielded 4 key findings: 1) asthma triggers included household mold, indoor smoking, pets, season change, strenuous exercise, extreme cold, and humidity; 2) social and educational support is lacking in Mi’kmaq communities despite a strong desire for these services; 3) cultural, linguistic, and geographic barriers to accessing support exist; and 4) family members are primary support resources.

**Conclusion:**

Improved support and educational resources are needed to foster effective Mi’kmaq asthma support networks. Future asthma interventions for marginalized populations must be culturally meaningful and linguistically accessible to those using and providing asthma support.

## Introduction

The health of Aboriginal peoples (First Nations, Inuit, and Métis), who comprise approximately 1.5% of the Canadian population, is poorer than that of non-Aboriginal Canadians by virtually all population-based measures of health and disease (1). Existing health inequalities facing Aboriginal peoples can be linked to social determinants of health at multiple scales: proximal (eg, poverty, overcrowded housing, smoking, household mold); intermediate (eg, health care systems, education systems, economic development, language), and distal (eg, colonization, racism, social exclusion, confinement to “Indian reserves,” mandatory attendance at Indian residential schools run by missionaries and colonial government agents) (2). New chronic conditions that have begun affecting Aboriginal peoples, especially type 2 diabetes, obesity, and cardiovascular disease, have received considerable attention from health researchers and policy makers (3). Although disparities in rates of these conditions and others (eg, suicide, HIV/AIDS, substance abuse) are well documented in the literature, albeit largely limited to the on-reserve population (4), little research addresses asthma despite its prevalence (5). Approximately 15% of Aboriginal youth in Canada aged 12 years and under have asthma (6). Fewer than half of those asthma-affected youth receive treatment (5); however, hospitalization rates for asthma among Aboriginal children have increased by 200% since the 1980s compared with a 50% increase among non-Aboriginal children (7,8).

The lasting effects of colonization that are known to affect asthma are easily evident in observations of the underlying conditions on Aboriginal reserves (9). Aboriginal peoples are more likely to live in overcrowded or substandard housing where respiratory infections resulting from exposure to dust, mold, and mildew can easily pass between family members (10). Smoking rates among Aboriginal peoples are nearly 3 times that of the general Canadian population (5,11). The use of woodburning stoves (often a financial necessity) and the practice of curing and tanning in living areas are common in Aboriginal communities, further contributing to degraded indoor air quality (12). Many Aboriginal peoples live in poverty (2), creating a financial barrier to asthma treatment and medication (13). Moreover, Aboriginal children are more likely to live with a single caregiver than Canadian youth in general, putting them at a greater risk of being exposed to a stressful home environment (5,11). Maternal stress during the first 7 years of a child’s life has been positively associated with an increased risk of asthma diagnosis (14). An understanding of the social determinants of health among Aboriginal peoples is necessary to an understanding of the social etiology of asthma in Aboriginal communities (2).

Although most asthma research is centered on physiologic aspects of the disease, less commonly examined are the psychosocial difficulties persistent among youth who have asthma. In addition to having the physiologic symptoms of asthma, youth with asthma are reported to experience low self-esteem, social isolation, family problems, poor relationships with peers, and worry (15,16). With satisfactory asthma-support programs in place, however, the quality of life for these young people can be greatly improved (17-19).

The research reported here is part of the first phase of a larger 3-phase, multisite national study that seeks to examine the support resources, support-seeking strategies, support and education needs, and intervention preferences of Aboriginal youth with asthma and their caregivers (referring to either biological parent or appointed guardian) to facilitate community-wide health-promoting behaviors. We employed a community-based participatory research (CBPR) design with guidance from regional community advisory committees (CACs) across 3 study sites in Canada (Alberta, Manitoba, and Nova Scotia) (20-22). To ensure that local considerations were prioritized from the inception of the research, each study site employed independent methods for project design, data collection, intervention design, and data analysis to tailor their studies to the common objectives. The focus of methods, results, and discussion in this article are limited to the data arising from the Nova Scotia site involving Mi’kmaq peoples, Aboriginal descendants of the original inhabitants of this region of Canada.

## Methods

The lead researcher for the Nova Scotia site (H.C.) spent a year (2010) establishing relationships with 5 Mi’kmaq communities in Unama’ki (Cape Breton), Nova Scotia (Figure) via each community’s health director. Once the research partnership was established, a local CAC was created with representation from each community’s cadre of health care professionals to offer guidance to the research team throughout the study. The CAC informed recruitment strategies, refined the research design, pilot-tested the data collection process, commented on preliminary findings, and communicated protocols to the communities. In spring 2011, 6 Mi’kmaq community researchers were hired and trained in participant recruitment and qualitative data collection.

**Figure:**
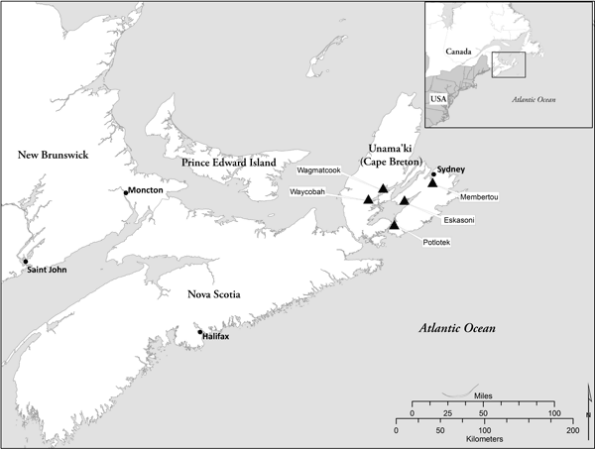
Map of Unama’ki (Cape Breton), Nova Scotia, showing location of 5 Mi’kmaq communities (Wagmatcook, Waycobah, Potlotek, Eskasoni, Membertou) participating in a study to identify gaps in asthma education, health promotion, and social support for Mi’kmaq families.

During May and June 2011, we recruited 17 Mi’kmaq families (17 caregivers and 21 youths) from the 5 communities by using a combination of purposive and snowball sampling techniques. Generally, a participating family consisted of 1 youth and 1 caregiver. In instances in which a caregiver was responsible for more than 1 youth with asthma, the family was defined as 1 caregiver and 2 (or more) youths. Inclusion criteria for families were 1) self-reported Mi’kmaq heritage, 2) caregiver-reported asthma or spirometry (lung function test) diagnosis of asthma and the use of any asthma medication in the last year, 3) age 8 to 12 years for youths with asthma, and 4) ability to understand and read English. Although all participants spoke English, we learned that many used Mi’kmaq in the home and in daily activities. Half of the community researchers were fluent or functionally conversant in both languages and, often spontaneously, conducted portions of their data collection activities in Mi’kmaq. All data were translated and transcribed in English.

The youth and their caregivers individually participated in semistructured interviews with the intent of identifying their asthma-related support needs and intervention preferences. The interviews with caregivers lasted approximately 1 hour each and were conducted by community researchers using an interview guide. (Interviews with youth were much briefer because they did not elaborate on their responses.) Open-ended questions addressed issues specific to existing community asthma support, asthma education, and asthma intervention preferences.

All participants were then invited to a 2-day asthma camp held in July 2011. The camp agenda included Aboriginal ceremonies (smudging, prayer); cultural activities (drum-making, drumming, singing, dancing); entertainment (games, art, relay races, movie night); social support (informal networking opportunities); and education (asthma awareness training, guest speakers with expertise in asthma support, education, and outreach). We also held 1 sharing circle and 1 focus group (23) during the camp with the 17 caregivers to help identify their asthma support intervention preferences. Distinct from focus groups, sharing circles emphasize equality among everyone involved in the circle. Sharing circles involve formal turn-taking versus spontaneous dialogue of a focus group; sacred meaning for many Aboriginal peoples in terms of spiritual and emotional growth; more sharing of the whole individual rather than just knowledge-sharing; nonjudgmental, helpful, and supportive discourse; respectful listening among participants; and, often, ceremony (eg, smudge, talking stick) (23). Both the focus group and sharing circle lasted approximately 1 hour each and were facilitated by community researchers who used an interview guide. Open-ended questions were used throughout these sessions. The sharing circle focused on both existing asthma support needs and intervention preferences of participants, while the focus group centered solely on intervention preferences. The interviews, sharing circle, and focus group were digitally recorded and transcribed to ensure accuracy. In addition to the participant data collected, the research team engaged in participant observation, recorded field notes, and held 2 debriefing sessions during the gathering, all of which were taken into consideration and applied to our analyses.

The research team tried out a sharing circle and focus group activity with participating youth, similar to those used with caregivers; however, these methods proved ineffective because the youth were uncomfortable with sharing personal experiences in a group. The flexible nature of CBPR use in the field enabled the research team to replace large group interactions with opportunities for the youth to make drawings of what it was like to have asthma. Then, in informal, individual, and small-group conversations, the youth talked about what they had drawn without interviewers using recording devices, which were also a source of discomfort for youth. As a result, the research team relied heavily on participant observation and field notes to document youth experiences with asthma.

All data obtained from the pre-camp interviews, participant observations, sharing circle, and focus group underwent a thematic analysis (24) to identify diverse factors influencing challenges facing Aboriginal youth with asthma and their families. This analysis offered suggestions for how youth with asthma and their caregivers can cope with the challenges.

Ethical approval for this study was obtained from the Dalhousie University Health Sciences Research Ethics Board and the Mi’kmaq Ethics Watch, an Aboriginal community-based ethics review board. Participating caregivers gave informed consent for their own and their child’s involvement in the study. In addition, youth participants assented to taking part in the research activities.

## Results

Four themes related to existing asthma support and future intervention preferences for Mi’kmaq families affected by asthma emerged from the data: 1) triggers and prevention strategies, 2) current asthma supports and services, 3) types of support-seeking strategies, and 4) desires for future support and education interventions and programs. The presence of these themes throughout the data was triangulated across the research team and CAC, increasing our confidence in the findings (25).

### Triggers and prevention strategies

Virtually all caregivers were aware of asthma triggers specific to their children and had developed strategies for avoiding them. Triggers were household mold, smoking, pets, season change, strenuous exercise, and extreme cold or humidity. Most caregivers indicated that they had had mold in their homes. Caregivers made extensive efforts to provide mold-free environments for their family. For example, one caregiver said: “We got this house . . . because [my son has] asthma . . . our last house we lived in had mold in the basement and it was causing him to get really sick.” Several caregivers also reported that smoking occurred throughout their communities either inside the home or in the car. Although smoking tended to be limited to certain rooms or floors of the residence, secondhand smoke still triggered attacks. Contact with household pets was also identified as an asthma trigger for some youth. In these cases, caregivers usually made an effort to remove the pet from the home in an attempt to more effectively manage their child’s asthma.

Virtually all caregivers developed strategies for avoiding triggers that they knew would influence the severity of their child’s asthma.

You have to do things differently in the house. You have to use certain products. Some products you find after a while are triggers, and one of the big things was floor wax. . . . You have to keep fresh air circulating in the house. But it is the opposite in the summer; when the [gravel] road is graded and the road is dusty, you have to keep the doors and windows closed. So you just always have to be aware of the triggers and of the air quality. [caregiver]

Although some caregivers used strategies that were developed on the basis of trial and error representing years of asthma management, others worked closely with their doctors to minimize the occurrence of asthma attacks.

[My daughter’s] asthma is induced by exercise. During her games she takes her inhalers when she gets sick. But we have learned this year to take the inhaler half an hour before the game and then play. This reduced her chance of getting an attack. That’s what the doctor told us to do. [caregiver]

Caregivers rely on lived experience and medical advice in formulating prevention strategies against asthma attacks. Although Mi’kmaq youth were often able to identify asthma triggers specific to their own condition, their awareness did not necessarily lead to avoidance. Despite recognizing strenuous exercise as a likely asthma trigger, 1 youth recalls, “I was at school and did the Terry Fox run and took an asthma attack. I forgot that I didn’t have my inhaler and was in the office for a half hour, waiting.” None of the youth were able to identify specific prevention strategies aside from acknowledging their reliance on inhalers. They also relied on their caregiver(s) to both supply and administer inhalers appropriately during an asthma attack.

### Current asthma supports and services

Participating caregivers reported a lack of community-level asthma support resources. When asked if they were aware of any asthma support resources or services in or outside their community, none were identified. As one caregiver explained, “Right now there is nothing. I never even heard of anything outside the community.” Geographic distance from services available in urban centers was cited as a major barrier to accessing support. Many caregivers shared their frustration with having to travel well outside of their reserve community to connect with specialized asthma support personnel.

Although Mi’kmaq youth did not specifically comment on the accessibility of asthma support resources and services in their community, several caregivers indicated that language barriers were contributing to existing asthma support gaps: “My kids are fluent in [our Aboriginal language] . . . and my youngest one, he is more comfortable speaking [our language] than English, so a lot of the times there’s that barrier of understanding” [caregiver]. Caregivers indicated that although health promotion materials were available for other chronic illnesses, very few of these were available in the Mi’kmaq language.

### Types of support-seeking strategies

Generally, caregivers rely on family members to provide them with the support they need to manage the demands of raising a child with asthma. Often these family members have direct experience raising their own child with asthma.

When [my children] are having an attack, it is really hard watching them suffering. Talking helps me cope. I have a really good support network with my sisters and my mother, family, and friends . . . you just don’t feel so alone and my sisters, 2 of them have kids with asthma, so it helps talking to them because they know what you’re going through. [caregiver]

Caregivers rely to a lesser extent on health professionals than family for asthma support resources. However, caregivers said such support usually involved writing prescriptions and giving basic instructions on administering medication rather than psychosocial support.

Participating youth also relied heavily on family, particularly mothers, as their primary asthma support. Teachers and peers also represent a support resource when children are absent from parental care. One youth explained, “. . . my parents and sometimes my teachers. If my teachers aren’t around then my friends.” However, not all youth indicated support beyond their immediate family, suggesting that many felt the same sense of social isolation reported in the literature (15,16).

### Desire for future support and education interventions

Many caregivers identified the need for increased information and educational resources. Caregivers felt they did not have enough information to manage their child’s asthma effectively.

My biggest wish is . . . if every health centre in [our region] would take 1 person and really seriously train them on this topic. So then they could pass on the information to the parents. Because a lot of the parents don’t have the proper information or they are not sure how to give the medication. That’s all I wish for. [caregiver]

Although doctors are acknowledged as asthma support providers, many caregivers feel they would benefit from additional education. One caregiver describes this scenario: “The doctor, like all she ever did was give him inhalers. That’s it. No information. I really don’t know anything about asthma right now.” Not only did caregivers want asthma education for themselves and their children; they also wanted the community to be better informed. Caregivers considered community education and provision of resources that promote community-wide understanding of asthma a priority. One caregiver described a situation where her daughter was involved in an after-school activity with another family: “When she came back, she was coughing, and her clothes smelled like smoke because the person who drove them was smoking in the car with them.”

In addition to increased education, caregivers also expressed a strong desire for support groups in their community. One caregiver explained, “We need support groups . . . I really don’t have anybody else to go [to] other than my doctor.”

Virtually all caregivers commented on the value of the asthma camp as a way of connecting with peers, specifically other Mi’kmaq caregivers dealing with asthma-related issues. They also suggested that future community-based health interventions and health-promoting resources be provided in the Mi’kmaq language.

Although participating youth were reluctant to identify any support or education needs, in several cases caregivers voiced concerns about a lack of asthma-friendly school policies: “[My daughter’s old school] didn’t want her to take [her inhaler]. They thought that she would get addicted to it. [Teachers don’t] like your child to take it when they need it, only when *they* [italics added] feel that the child should take it.” Caregivers suggested that culturally relevant, school-based, asthma support is needed for youth with asthma to effectively manage their condition; school teachers, counselors, administrators, and teaching assistants needed asthma-awareness training.

## Discussion

The lack of culturally relevant and linguistically accessible asthma support resources is problematic for the Mi’kmaq youth and caregivers involved in this study. Adequate support resources can improve the quality of life for asthma-affected youth; conversely, the absence of these supports can lead to ineffective asthma management, resulting in harmful health consequences (15-17). Our study results suggest there is a need and desire among Mi’kmaq families in this region for a community-level asthma-support intervention. For future intervention strategies to be effective, however, the issue of accessibility must be addressed. Because these Mi’kmaq communities are geographically removed from each other (ranging from 15 to 80 miles apart), it is imperative that future interventions be implemented within each community. Providing culturally relevant and linguistically accessible resources will also honor Mi’kmaq traditions, values, and language (26,27).

Given that many Aboriginal communities embrace a philosophy of a community-raised child (28) in which family members, teachers, and peers are all looked upon to provide support for youth with asthma and their caregivers, it is paramount that entire communities and government agencies support asthma management strategies (eg, reducing traffic-related dust on gravel roads, eliminating mold from housing, relieving overcrowding in housing). Improving asthma education at both the family and community level would result in more effective asthma management for Mi’kmaq youth with asthma. Knowledge holders such as health professionals must be made aware of the lack of asthma-related information available to community members and must be willing to provide it through a medium that is culturally relevant, accessible, and designed for a broad audience. The results of this study indicate the need for expanded awareness of asthma: what triggers asthma attacks, strategies for avoiding attacks, methods of administering asthma medicine, and an overall improved understanding of the condition. In addition, greater consistency between school policy and asthma management needs is required.

Caregivers expressed strong interest in establishing support groups. Communities fostering socially supportive environments are healthier than their socially unsupportive counterparts (29). Asthma support groups or support networks are a viable, culturally appropriate way to supplement asthma education strategies. Regular in-community gatherings of asthma-affected families would provide caregivers and youth with a safe space to talk about asthma, share information, voice concerns, and ask questions in an effort to aid each other in their own asthma management. In our case, the multigenerational asthma camp was an effective strategy for connecting Mi’kmaq families with their peers in the region.

Although our study provides insight into the asthma support needs and preferences of on-reserve Mi’kmaq youth with asthma and their caregivers in 5 Mi’kmaq communities in Unama’ki (Cape Breton) Nova Scotia, its geographic and cultural applicability beyond these communities is limited. Nevertheless, the study reveals a lack of asthma support resources in these communities. Improved support and educational resources are needed to foster effective asthma support networks that include both people closely involved with youth with asthma and members of the larger community. Although the general public faces many of the same challenges reported here in terms of asthma support and education (15-17), the social determinants of Aboriginal peoples’ health exacerbate existing health inequalities for this group. Asthma support and education specific to Aboriginal peoples are required for effective management of this disease (2). Future asthma support interventions involving Aboriginal peoples or other marginalized populations must overcome geographic barriers, be culturally relevant, and be linguistically accessible both to those using asthma support and to those providing it.
