# Erratum, Vol. 9, December 27 Release

**DOI:** 10.5888/pcd10.120039e

**Published:** 2013-06-27

In the article “Identifying Gaps in Asthma Education, Health Promotion, and Social Support for Mi’kmaq Families in Unama’ki (Cape Breton), Nova Scotia, Canada,” an error occurs in the first sentence of the Introduction. That sentence should read, “The health of Canada’s 1.5 million Aboriginal peoples (First Nations, Inuit, and Métis) (http://www.statcan.gc.ca/pub/89-645-x/2010001/count-pop-denombrement-eng.htm) is poorer than that of non-Aboriginal Canadians by virtually all population-based measures of health and disease (1).”

The correction was made to our website on June 19, 2013 and appears online at http://www.cdc.gov/pcd/issues/2013/12_0039.htm. We regret any confusion or inconvenience this error may have caused.

